# The Role of microRNAs in Inflammatory Bowel Disease

**DOI:** 10.3390/ijms26104750

**Published:** 2025-05-15

**Authors:** Aneta Sokal-Dembowska, Sara Jarmakiewicz-Czaja, Kacper Helma, Rafał Filip

**Affiliations:** 1 Faculty of Health Sciences and Psychology, Collegium Medicum, University of Rzeszów, 35-959 Rzeszów, Poland; 2 Department of Internal Medicine, Faculty of Medicine, Collegium Medicum, University of Rzeszow, 35-959 Rzeszow, Poland; 3 Gastroenterology Clinic, Center for Comprehensive Treatment of Inflammatory, Bowel Disease Regional Hospital No. 2 in Rzeszow, 35-301 Rzeszow, Poland

**Keywords:** Crohn’s disease, inflammatory bowel disease, microRNA, ulcerative colitis

## Abstract

Deregulation of microRNAs (miRNAs) has been implicated in the development of inflammatory bowel disease (IBD). Specific miRNAs are differentially expressed in patients with IBD compared to healthy individuals. Regulation of their expression can modulate the inflammatory response, the composition of the intestinal microbiota, and intestinal barrier function. miRNAs can regulate the immune and inflammatory response via multiple mechanisms, from Th1/Th17 regulation and ferroptosis to modulation of NLRP3 (NOD-like receptor family, pyrin domain-containing 3) and control of the NF-κB (nuclear factor kappa-light-chain-enhancer of activated B cells) pathway. The use of miRNAs as biomarkers and therapeutic targets may help monitor IBD treatment and support the development of new, more individualized therapies that minimize common side effects.

## 1. Introduction

Inflammatory bowel diseases (IBDs) are a group of idiopathic conditions characterized by localized inflammation and ulceration of the gastrointestinal wall. IBD has a chronic character, with periods of symptom exacerbation alternating with remission. It is a highly variable condition, with symptoms ranging from mild to debilitating, and severity varying from mild to severe [1] The exact mechanism of IBD development remains unclear, but it has been suggested to result from a complex interplay between genetic predisposition, impaired anabolic response, exposure to various environmental factors, changes in the gut microbiome, dysfunction of the intestinal barrier, and immune system dysregulation [2,3]. Dysbiosis of the gut microbiota is observed in both forms of IBD. It can be caused by various environmental factors, such as diet or infections, but also by genetic factors that independently increase the risk of IBD. Dysbiosis itself may contribute to increased inflammation, while pre-existing inflammation can disrupt microbiota composition, indicating the importance of inflammation in IBD progression. These factors interact in complex and dynamic ways. Genetic susceptibility can potentially influence immune response, while environmental exposures can modulate the gut microbiome, leading to epigenetic changes that further influence disease progression [4]. These interconnected mechanisms act through several key molecular pathways involved in the pathogenesis of IBD, such as nuclear factor kappa-light-chain-enhancer of activated B cells (NF-κB), Janus kinase 1 (JAK1), Signal transducer and activator of transcription 3 (STAT3), Toll-like receptor 4 (TLR4), mammalian target of rapamycin complex 1 (mTORC1), and interleukin signaling pathways including interleukin (IL)-17 and IL-23, all contributing to immune dysregulation, epithelial barrier disruption, and chronic intestinal inflammation [5]. A number of microRNAs (miRNAs) that are associated with IBD have been identified [6]. miRNAs are small, single-stranded endogenous noncoding RNAs that originate from longer primary miRNA (pri-miRNA) transcripts, which are transcribed from miRNA genes [7]. The mature form of miRNA usually ranges from 18 to 24 nucleotides in length [8]. A considerable amount of evidence indicates that miRNAs are involved in the regulation of inflammation, immune response, and regulation of microbiota, which in turn play important roles in the pathophysiology of IBD [6]. This makes understanding the role of miRNAs in the regulation of inflammatory processes in IBD necessary to fully understand the mechanisms of IBD development and progression. The objective of this review is to summarize the current knowledge on the mechanisms of miRNAs’ action in IBD, their role in regulating inflammatory pathways in IBD, and the potential of specific miRNAs to serve as biomarkers in this disease.

## 2. Inflammatory Bowel Diseases

The two most common subtypes of IBD are Crohn’s disease (CD) and ulcerative colitis (UC) [1]. CD is characterized by granulomatous inflammation, which can occur throughout the gastrointestinal tract and involve all layers of the intestinal wall, promoting strictures and fistulas. UC can affect all areas of the colon and mainly affects the superficial mucosal layer of the large intestine. The lesions are superficial, which promotes the formation of erosions and ulcers [9]. Typical symptoms of CD include abdominal pain, diarrhea, weight loss, and fever, while typical symptoms of UC are abdominal pain, bloody diarrhea, fecal incontinence, and fatigue. Symptoms may change over time [10,11].

IBD, as a chronic and progressive condition, can lead to the development of serious complications, both in the gastrointestinal tract and in other organs. Some patients with UC develop enteropathic arthritis, ankylosing spondylitis, primary sclerosing cholangitis (PSC), anemia, inflammatory skin conditions, and inflammatory eye conditions [10,11]. Patients suffering from IBD have a higher risk of developing colorectal cancer (CRC), which is linked to dysplastic changes in the colonic mucosa. This risk is higher in patients with UC and increases over time after diagnosis [12,13]. UC patients with concomitant PSC are at increased risk of developing cholangiocarcinoma (CCA) [12,14]. Individuals with perianal or rectal CD are at higher risk for rectal cancer, especially fistula-related adenocarcinoma. IBD patients, in general, also have a higher risk for non-gastrointestinal solid-organ tumors and hematological malignancies [12]. One of the most serious long-term complications of IBD is intestinal fibrosis, which is a pathological, uncontrolled repair process of intestinal tissues resulting from dysregulation of regenerative mechanisms. It is estimated that it can affect more than 50% of patients with CD [15]. Intestinal fibrosis is less common in the course of UC. In this process, there is excessive deposition of extracellular matrix (ECM) by fibroblasts in the intestinal wall, which in turn leads to its thickening and hardening. This can lead to loss of intestinal elasticity and function, which in turn leads to the formation of strictures and obstructions [15,16,17]. Intestinal strictures are caused by excessive accumulation of scar tissue. They occur in about 5 to 10% of UC patients and can lead to obstruction. Persistent inflammation can lead to fistulas, which develop in 20% to 40% of CD patients and may contribute to diarrhea and malabsorption [18]. Intestinal fibrosis usually develops in sections of the intestine affected by inflammation, but it is worth noting that inflammation alone is not the only factor causing fibrosis. It has been proven that suppression of inflammation alone is not sufficient to prevent fibrosis, and therefore, it has been suggested that there are other, independent mechanisms of fibrosis [16,19]. Factors leading to intestinal fibrosis include: imbalances of matrix metalloproteinases (MMPs) and tissue inhibitors of metalloproteinases (TIMPs), macrophage dysfunction, and mitochondrial dysfunction [20].

According to Zhao et al., the prevalence of CD in Europe ranges from 1.5 to 331 per 100,000, while the prevalence of UC ranges from 2.4 to 431 per 100,000 [21]. In the United States, the overall prevalence of IBD is estimated to be 812 per 100,000 [22]. Recent epidemiological data from the Global Burden of Diseases suggest that IBD prevalence and incidence rates are constantly increasing. It is estimated that between 1990 and 2021, the global incidence of IBD increased by 88.3% [23]. Most people are diagnosed with UC between the ages of 20 to 30 and 50 to 80, and with CD between the ages of 15 to 25, but all ages can be affected [10,11]. These data indicate that IBD is becoming an increasingly serious public health problem, requiring further research into more effective diagnostic and therapeutic strategies, especially considering that the exact etiology and pathophysiology of IBD are still unknown.

## 3. MicroRNA Function and Synthesis

The first miRNA to be discovered was lin-4 RNA, which was identified in the nematode *Caenorhabditis elegans* by Lee et al. in 1993 [24]. Since then, thousands of miRNAs have been identified, some of which are associated with the pathogenesis of IBD. miRNAs are fundamental modulators of gene expression, modulating it through epigenetic and post-transcriptional mechanisms. miRNAs influence protein production by binding to mRNA, resulting in mRNA degradation or post-transcriptional gene silencing (PTGS). miRNAs have been shown to be involved in almost every cellular process, and a single miRNA can regulate many different genes or even entire cellular pathways. On the other hand, one gene can be regulated by several different miRNAs, which makes the relationship between miRNAs and individual diseases very complex [7].

**Figure 1:**
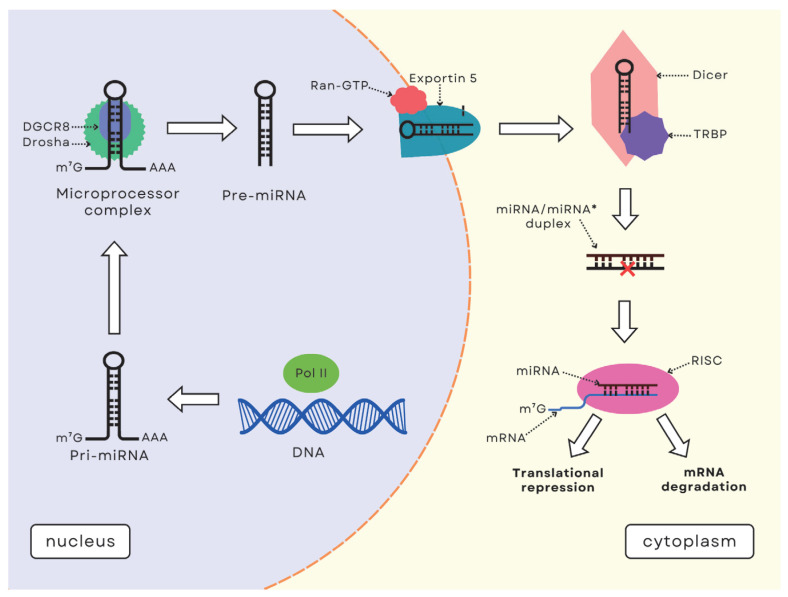
General scheme of miRNA biogenesis and mechanism of action. Abbreviations: Pri-miRNA—primary miRNA; DGCR8—DiGeorge syndrome critical region gene 8; pre-miRNA—precursor-miRNA; TRBP—transactivation-responsive RNA-binding protein; miRNA—microRNA; mRNA—messenger RNA; RISC—RNA-induced silencing complex; miRNA* (passenger strand)—the second strand in the miRNA/miRNA* duplex, which is typically discarded. The red X indicates the degradation of the miRNA* strand.

Deregulation of miRNAs is associated with many diseases, including IBD, and can lead to disorders when imbalanced [25]. This highlights the importance of understanding miRNA biogenesis and its regulatory mechanisms because disruptions in these processes can contribute to disease development. The miRNA biogenesis and mechanism of action involve multiple steps (Figure 1). It begins in the nucleus, where miRNA genes are transcribed by RNA polymerase II (Pol II) into pri-miRNA. This is followed by nuclear cleavage, in which the pri-miRNA is cleaved into shorter precursor-miRNA (pre-miRNA) by the Microprocessor complex, which includes the Drosha enzyme and its cofactor DiGeorge syndrome critical region gene 8 (DGCR8) [26]. The pre-miRNA is then transported from the nucleus to the cytoplasm by exportin-5 (XPO5) in the presence of Ran-GTP, and further processed by Dicer, a type III cytoplasmic endoribonuclease [26,27,28]. Dicer carries out pre-miRNA maturation by binding to pre-miRNA and cleaving it into a miRNA/miRNA* duplex, from which the mature miRNA guide strand is loaded into the RNA-induced silencing complex (RISC). This miRNA strand guides RISC by binding to target messenger RNAs (mRNAs) through the Argonaute (AGO) protein, leading to gene silencing [26,27]. The majority of miRNAs suppress target mRNA expression by binding to specific sequence motifs in the 3′-untranslated region (3′-UTR). miRNA can also interact with other regions, such as the 5′-untranslated region (5′-UTR), gene promoters, and coding sequences [8,29]. Gene suppression occurs through either mRNA translational repression or mRNA degradation, and mainly occurs within processing bodies (P-bodies) [26,27].

## 4. The Role of miRNAs in the Immunological Response and Regulation of Inflammation in IBD

The target genes of miRNAs are responsible for controlling inflammation and the immune response. Genes targeted by overexpressed miRNAs in IBD show significant convergence towards common pathways involved in adaptive immune responses, interleukin signaling, cytokine activity, and inflammation [30]. miRNAs affect the development, differentiation, and apoptosis of various immune cell populations and mediate the control of innate and acquired immunity [31]. They modulate numerous intracellular signaling pathways involved in both pro-inflammatory (e.g., miR-2, miR-124) and anti-inflammatory (e.g., miR-10a, miR-141, miR-320) responses, playing a key role in the pathogenesis of IBD. They are also involved in the regulation of the epithelial barrier, and their effects can be twofold. They can weaken the intestinal barrier (e.g., miR-21, miR-122a, miR-191a, miR-212, miR-675, miR-874) or strengthen it (e.g., miR-93, miR-200b) [32]. A compromised intestinal barrier is known to increase antigen penetration, which significantly contributes to intestinal inflammation [33].

### 4.1. microRNAs in Th1/Th17 Regulation

Th1/Th17 cell-dependent inflammatory responses play an important role in the pathogenesis of IBD [34,35]. Li et al. demonstrated that miR-374b-5p and miR-106a-5p may play a transient role in the regulation of inflammatory responses in IBD, and miRNA-374b-5p can further enhance Th1 and Th17 cell differentiation. The levels of these miRNAs were significantly elevated in patients with CD and UC. These miRNAs may contribute to the development of IBD by regulating IL-10/STAT3 signal transduction [36]. In turn, miR-219a-5p suppresses intestinal inflammation by inhibiting the immune responses mediated by Th1/Th17 [37].

### 4.2. miRNAs in Ferroptosis and Its Impact on Cell Death in IBD

An association between miR-129-5p contained in exosomes derived from umbilical cord mesenchymal stem cells (hucMSC-Ex) and inhibition of ferroptosis has also been demonstrated. In an in vivo study, hucMSC-Ex enriched with miR-129-5p can inhibit ferroptosis. hucMSC-Ex alleviates IBD by acting on Acyl-CoA synthetase long-chain family member 4 (ACSL4) via miR-129-5p, leading to reduced levels of lipid peroxidation and ferroptosis, reduced inflammation, and damage repair [38]. Ferroptosis, a type of regulated cell death, has been implicated in the development of IBD. It is characterized by excessive accumulation of reactive oxygen species and lipid peroxidation in cells. Ferroptosis may lead to the development of IBD as a result of damage to intestinal epithelial cells (IECs) and the mucosal barrier [39].

miRNAs are also responsible for the regulation of autophagy in the cell via various processes. Autophagy has a protective function against stressors, especially oxidative stress, endoplasmic reticulum stress, or various intracellular pathogens (xenophagy process) [40]. Autophagy maintains homeostasis in epithelial cells, Paneth cells, and immune cells, and its dysfunction can lead to the development of IBD [41]. Autophagy protects the intestinal mucosal barrier, mainly by regulating cytokines and modulating apoptosis [42]. Reduced miR-192-5p expression in inflamed intestinal tissues correlated with impaired intestinal epithelial barrier (IEB) function, while overexpression of miR-192-5p alleviated TNF-induced IEB dysfunction by targeting Rictor (Rapamycin-insensitive companion of mTOR), which increased autophagy flow in enterocytes [43]. mTOR interacts with specific adaptor proteins and forms two complexes, mTOR complex 1 (mTORC1) and mTOR complex 2 (mTORC2), consisting of mTOR, mLST8, and Rictor [44]. Rictor/mTORC2 signaling regulates apoptosis in intestinal epithelial cells during colitis and may prevent damage to the IEB [45].

### 4.3. miRNAs in Modulation of the NLRP3 Inflammasome and Alleviation of IBD

Due to their immunoregulatory function, human hucMSC-Ex have become a new research target in the search for an effective therapy for IBD [46]. In a study by Cai et al., exosomes derived from hucMSC cells carrying miR-378a-5p inhibited NLRP3 (NOD-like receptor family, pyrin domain-containing 3) inflammasomes in macrophages and prevented pyroptosis, thereby contributing to the alleviation of IBD symptoms [47]. Studies indicate a dual effect of NLRP3 in IBD. On the one hand, its activation in the early stages of the disease may benefit the integrity of the intestinal epithelium, promoting its repair and mucosal regeneration [48]. On the other hand, activation of the NLRP3 inflammasome can initiate inflammatory processes, affecting intestinal epithelial cells and macrophages, leading to tissue damage and the development of IBD [49,50]. Thus, increased NLRP3 inflammasome activity appears to be a key step in the initiation of inflammation and the development of clinical symptoms in IBD [47]. In another study, Scalavino et al. showed that miR-369-3p was able to modulate the expression of the inflammasomes BRCC3 (BRCA1/BRCA2-containing complex subunit 3) and NLRP3. Transfection with the miR-369-3p mimetic was able to reduce NLRP3 expression by modulating BRCC3 following inflammasome activation. These findings suggest that miR-369-3p may alleviate inflammation in IBD patients by modulating the NLRP3 inflammasome complex [51].

### 4.4. miRNAs in the Regulation of the NF-κB Pathway

miRNAs also control the NF-κB signaling pathway in IBD [52]. NF-κB modulates inflammation through multiple interdependent transcriptional and post-transcriptional processes [53]. Feng et al. reported that miR-149-3p may act as a suppressor in colitis by negatively regulating NF-κB activation and increasing AMP-activated protein kinase (AMPK) activation. Deletion of miR-149-3p increased IκBα phosphorylation and inflammatory responses in vivo. In contrast, miR-149-3p mimetics decreased IκBα phosphorylation, NF-κB transactivation, and some inflammatory cytokines (Il-6, Il-17) mediated by NF-κB in vitro [54]. Inhibition of the NF-κB pathway and release of pro-inflammatory cytokines by miR-497 in a DSS (dextran sulfate sodium)-induced IBD mouse model and in RAW264.7 cells led to inhibition of inflammation [55]. Chronic and systemic inflammation in IBD can lead to a range of complications, including extra-intestinal manifestations [56]. The release of exosomes from hAESCs (amniotic epithelial stem cells) enriched with microRNA-23a-3p post-transcriptionally decreased TNFR1 (tumor necrosis factor receptor 1) expression, attenuating the NF-κB factor signaling pathway in colorectal epithelial cells after exposure to DSS [57].

miRNAs play an important role in the regulation of the immune response and inflammation in IBD, affecting various IL-10/STAT3, NF-Κb signaling pathways, Th1/Th17 regulation, and modulation of the NLRP3 inflammasome. miRNAs can both promote and suppress inflammatory processes by modulating immune cell and epithelial barrier activity, making them potential therapeutic targets for the treatment of IBD.

### 4.5. miRNAs in the Regulation of Intestinal Microbiota and Epithelial Barrier Function

In the course of IBD, the immune response against the gut microbiota is exacerbated. The gut microbiota of patients with IBD is characterized by reduced diversity and reduced community stability. There is a predominance of *Ruminococcus gnavus*, *Escherichia coli,* or *Bacteroidetes,* and a reduction in *Firmicutes* clusters [58]. The results of current studies emphasize the importance of the interaction between miRNA and the gut microbiota. This communication can go in one or both directions. It has been shown that individual miRNAs can promote bacterial growth, modulate inflammation associated with dysbiosis, and some bacteria can influence their expression, which alters the immune response and contributes to the protection of intestinal barrier function. Therefore, miRNA dysregulation or deficiency may be associated with changes in the microbiota, disruption of the intestinal barrier, and an excessive immune response [8]. It has been proven that there is an association between specific miRNA expressions and key gut bacteria at different stages of CD development, confirming their important role as potential molecular biomarkers [59].

### 4.6. miRNAs in the Regulation of Fibrosis and Intestinal Homeostasis

miRNAs exhibit both antifibrotic and profibrotic effects, but their exact role in the development of fibrosis requires further investigation [60]. It has been suggested that in CD, low levels of miR-29 may have a profibrotic effect, while miRNAs within the miR-200 family have the potential to act protectively in the development of intestinal fibrosis [61]. Additionally, in the study by Li et al., miR-155 levels were higher in the intestinal tissues of CD patients with present strictures compared to those without fibrotic strictures, suggesting that miR-155 could play a role in the development of intestinal fibrosis [62].

Deletion of miR-149-3p in mice altered the composition of the gut microbiota. miR-149-3p -/- mice had increased relative abundance of inflammation-associated gut microbiota and decreased relative abundance of beneficial microorganisms. These changes increased susceptibility to DSS-induced colitis. Both miR-149-5p and miR-149-3p suppressed the colonic inflammatory response in vitro and in vivo [54]. In the study by Luo and Chen, miR-149-3p and miR-149-5p were significantly reduced in inflamed tissues from patients with CD and UC. It is likely that reduced miR-149-3p and miR-149-5p expression is associated with disease activity in IBD patients [63].

The transcription factor cAMP-responsive element-binding protein H (CREBH), miR-143/145, and the insulin-like growth factor (IGF) system are key mediators in the communication between host intestinal homeostasis and gut bacteria. In a study by Wade et al., increased colonization of *Akkermansia muciniphila* in the intestine of CREBH gene-deficient mice improved intestinal CREBH expression, which led to alleviation of endoplasmic reticulum stress, improved tight junctions, and promoted IEC proliferation. This suggests that reduced levels of *Akkermansia muciniphila* may promote the development of IBD [64].

Diet-induced intestinal dysbiosis may lead to IBD-associated epigenetic changes, which are associated with a decrease in miR-148a, miR-152, and miR-143/145a in colonocytes. Thus, a high-fat diet rich in simple sugars may alter the miRNA profile of visceral fat exosomes, and alcohol consumption has been associated with increased miR-122 and miR-155 expression and increased intestinal permeability [65]. Dysbiosis can also stimulate immune cells to release IL-1β, which can lead to tissue damage and inflammation in IBD [66].

In the Casado-Bedmar study, in vivo inhibition of endogenous miRNAs (let-7b and miR-21) significantly improved colonic inflammation, intestinal mucosal barriers, and dysbiosis. miR-21 had a greater effect on intestinal barrier function, permeability, and permeation of bacterial components. In contrast, let-7b had a stronger effect on modulating the gut microbiota [67]. Interestingly, Li et al. observed that delivery of exogenous miR-2911 extracted from vicuña can also modulate the intestinal microbiota, reducing *Escherichia-Shigella* abundance and alleviating colitis symptoms [68].

Interactions between miRNAs and the gut microbiota play a key role in regulating the immune response and intestinal barrier function. Dysregulation can lead to changes in the composition of the microbiota, disruption of the intestinal barrier, and an exaggerated immune response.

Table 1 summarizes the mechanisms by which specific miRNAs may modulate the immune system response, intestinal barrier integrity, and inflammation in IBD.

## 5. MicroRNAs as Biomarkers of Inflammatory Bowel Diseases

miRNAs can be used as biomarkers to assess CD or UC disease activity. Atanassova et al. observed that changes in miRNA expression can be associated with both the severity of disease activity and the distinction between IBD exacerbation and remission phases. In a study involving 70 IBD patients, they linked increased serum miR-16 expression to disease activity and severity. In addition, they point to the association of miR-16 with the penetrating and constricting phenotype [69]. In another study, Cordes et al. observed elevated serum miR-320a levels in CD patients during the active phase of the disease compared to patients in remission. In addition, after evaluating CD disease activity indices (CDAI), the researchers noted that patients with mild CD disease activity (CDAI 150-220) showed lower levels of miR-320a compared to patients with more severe CD activity (CDAI > 220). Such potential in association with miR-320a was not observed in UC patients [70]. In turn, Wang et al. linked higher miR-223 expression in IBD patients compared to controls. In addition, they noted, based on the CDAI index in CD and the Mayo scale in UC, that miR-223 was also correlated with disease severity [71]. Wu et al. also showed that the expression of individual miRNAs can vary in different sections of the intestine in CD [72]. Fasseu et al. demonstrated disease- and stage-dependent changes in miRNA expression. In both CD and UC, miR-26a, miR-29b, miR-126, miR-127-3p, and miR-324-3p were overexpressed in inflammatory tissues in patients [73]. In contrast, Dalal et al. point to the problem of dysplasia associated with chronic inflammation. They pay particular attention to miR-31, whose expression was increased in both patients with IBD-induced dysplasia and patients with IBD-associated CRC. To evaluate dysplasia, it may be particularly helpful to examine miRNA expression levels [74]. miRNAs are known to regulate key oncogenic processes in CRC, such as proliferation, angiogenesis, apoptosis, and autophagy [75]. In addition, IBD patients, especially those with UC and concomitant PSC, are at increased risk of developing cholangiocarcinoma (CCA). Several miRNAs were found to be dysregulated in CCA and associated with risk factors related to CCA [12,14,76]. In a meta-analysis, Liu et al. evaluated the occurrence of correlations between polymorphisms in different types of miRNAs and the incidence of IBD. They suggest that the miR-146a polymorphism was significantly correlated with susceptibility to IBD, particularly UC. They explain that the polymorphism of the miR-146a gene mapped to chromosome 5q33 can, for example, reduce the expression of interleukin-1 receptor-related kinase-1 and tumor necrosis factor 6 receptor-related factor 6 [77]. Iborra et al. identify six miRNAs that differ in patients with active CD and those in remission (miR-188-5p, miR-877, miR-145, miR-140-5p, miR-128, miR-18a). In addition, they compared miRNAs among UC patients and observed 15 changes in miRNA expression between patients in the active phase of the disease and in remission, but the results were not statistically significant. In addition, they compared miRNAs in patients with CD and UC to a control group. The results indicated that there were 6 differences between CD and healthy subjects and 25 differences between UC and healthy subjects. They also indicated the overexpression of 12 different miRNAs from the serum, occurring in both CD and UC (miR-127-3p, miR-491-5p, miR-18a, miR-145, niech-7b, miR-185, miR-29c, miR-19b, miR-20b, miR-106a, miR-17, and miR-222) [78]. In contrast, Zahm et al. looked for specific miRNAs in pediatric CD. They observed significant changes in 24 miRNAs that were upregulated. For 11 miRNAs, they indicate that they may be promising diagnostic agents as non-invasive biomarkers in pediatric CD [79]. Moreover, to examine the association of miRNAs with pediatric CD, Lv et al. also determined correlations with the gut microbiota. They noted a linkage to eight tissue miRNAs (hsa-miR-215-5p, hsa-miR-12135, hsa-miR-194-5p, hsa-miR-509-3-5p, hsa-miR-4448, hsa-miR-212-5p, hsa-miR-501-3p, and hsa-miR-503-5p) and seven gut microbes (*S. enterica*, *D. raffinosedens*, *R. intestinalis*, *T. metallivorans*, *Dorea sp. AGR2135*, *S. sonnei*, and *E. coli*). They found that changes in the expression of individual miRNAs can affect changes in the composition of the gut microbiota [59]. In another paper, researchers point to five types of miRNAs with changes in expression (miR-126-5p, miR-4433b-5p, let-7d-5p, miR-3121-5p, and miR-221-5p) which may predispose individuals to CD itself, as they are involved in regulating inflammation [80]. Mohammadi et al. studied the differential expression of miRNAs in different sections of the gastrointestinal tract in patients with CD compared to controls. Based on their results, they indicate that tissue overexpression of miRNAs may be related to the location of inflammation and age [81]. Yarani et al. sought to identify similarities in changes in miRNA expression from both CD and UC based on a review of the literature. They noted that there is increased expression of miR-146a-5p, miR-223-3p, miR-21-5p, and miR-31-5p in colon tissues, while from blood serum, differences in expression in both phenotypes were observed in miR-223-3p, miR-16-5p, miR-30e-5p, miR-142-5p, miR-199a-5p, and miR-362-3p [6]. In addition, Chen et al. examined miRNA expression in CD, including patients with NOD2 genotypes. They noted that NOD2-dependent induction of miR-155 in CD patients may vary depending on the cell type and nature of the disease. However, expression was reduced after treatment with muramyl dipeptide [82]. In another study, Caparrós et al. showed an association with differential expression of miR-20a-5p, miR-376a-3p, tested from the peripheral blood of CD patients, compared to controls. In addition, they observed a correlation between the expression of the previously mentioned miRNAs and the levels of pro-inflammatory cytokines [83]. In a paper by Yarani et al., the authors conclude that there is an association of 28 miRNAs that could be used for IBD diagnostic purposes [6].

## 6. Micro-RNAs as Therapy and Predictors of Response to Applied Treatment in Inflammatory Bowel Diseases

Some researchers indicated that changes in the expression of particular miRNAs observed in CD or UC could serve as potential predictors of therapy efficacy. One group of drugs for which miRNAs could serve as predictors of therapy is anti-TNF. Based on an in-depth bioinformatics analysis, Pu et al. developed a predictive model for the response to applied Infliximab (IFX) therapy in UC patients. They identified 15 potential miRNAs that could serve in such an assessment [84]. In another study, Casertano et al. observed reduced expression of miR-20a, miR-126 in both serum and feces in children with CD who received IFX therapy. The miRNA changes were correlated with the Paediatric Crohn’s disease activity index (PDCAI) and C-reactive protein (CRP) [85]. However, the intestinal tissue in CD is variable for miRNAs, so Cervera-Seco et al. point to the need to design studies that determine treatment effects based on miRNAs in IBD [86]. In addition, using the expression of miR-126, Let-7e, which are involved in the regulation of the immune system, it is possible to predict clinical remission in CD patients taking biologic therapy [87]. Heier et al. identified three miRNAs that could potentially be used as predictive markers in evaluating the efficacy of anti-TNF and glucocorticoids (GC) treatment [88]. Another study evaluated the miRNA expression of the use of anti-TNF or glucocorticosteroid treatment in pediatric patients with IBD. The authors indicate that five types of miRNAs (let-7c, miR-126, miR-146a, miR-320a, and miR-146b) could potentially monitor treatment efficacy [89]. On the other hand, Luo et al. included 94 UC patients, whom they divided into three GC-treated groups (standard-dose GC, GC resistance, and GC sensitivity). They observed six potential types of miRNAs (miR-30e-3p, miR-16-2-3p, miR-642a-5p, miR-32-5p, miR-224-5p, and miR-150-5p) that could be used to assess GC resistance in UC patients [90]. In addition, in the case of UC, Sáez-González et al. observed that five miRNAs changed expression after granulocyte and monocyte apheresis [91].

miRNAs show potential not only in evaluating the effectiveness of the treatment applied, but can also take an active role in therapy [92]. Examples include miR-2, which has been shown to down-regulate the pro-inflammatory IL-23 (interleukin-23) response, or miR-657 that inhibits TNFα receptor and NF-κB signaling [93,94]. Fang et al. point to the potential therapeutic potential of miR-31-3p mimetics in colitis [95]. In addition, miR-291 has shown the ability to transport to intestinal bacteria, reducing Escherichia-Shigella [68]. Another example is miR-129-5p, which inhibits ferroptosis and lipid peroxidation, resulting in reduced inflammation in the gut [38]. Liao et al. observed that miR-195a-3p from Treg-exo can exert effects on caspase 12, and as a result, promote restoration of IEB damage [96]. However, Aggeletopoulou et al. describe that altering miRNA activity can exert effects on many other signaling pathways and target genes, which can generate previously unknown side effects [97]. Innocenti et al., on the other hand, point out that the therapeutic value would need to be determined for its effect on reducing inflammation or predisposing individuals to intestinal fibrosis [98].

## 7. Potential Directions for Research and Clinical Applications

Discoveries related to miRNAs may support the development of new therapeutic approaches that target interactions between host genes, the microbiome, and its bioactive components [64]. As miRNAs can modulate the expression of pro-inflammatory genes and regulate ferroptosis and tissue damage, it appears that specific miRNAs may be therapeutic targets for IBD [38]. Their use may allow the regulation of only those elements of the inflammatory process that are disturbed. Development of therapies based on small molecules or molecules that can modulate miRNA expression may allow precise control of inflammatory responses in the gut. They can either inhibit an excessive inflammatory response or support anti-inflammatory mechanisms. The use of “personalized” therapies may not only bring benefits in terms of therapies, but also reduce the risk of side effects in this group of patients.

To date, most data on miRNA-based therapeutic strategies in IBD have come from preclinical studies. These include direct administration of specific miRNAs or their mimetics, such as miR-602, miR-146b, osa-miR164d (derived from ginger), and miR-26a, which have shown anti-inflammatory effects in animal models [99,100,101,102].

Despite promising outcomes, the clinical translation of miRNA-based therapies faces several significant challenges. These include difficulties in precisely delivering therapeutics to inflamed tissues, ensuring their stability in the body, and achieving efficient cellular uptake [97]. Additional obstacles arise from inter-patient variability, such as disease phenotype, differences in gut microbiota composition, degree of epithelial barrier damage, or deregulation of the immune response. Another major limitation is the lack of a suitable biocompatible and biodegradable carrier system that can ensure safe and targeted delivery of miRNAs [103]. It is also important to develop reliable biomarkers that would not only facilitate the design of personalized therapies but also help predict patients’ response to treatment [104]. Future studies should focus on the development of safe and effective miRNA delivery systems, as well as the development of in vitro intestinal and organoid models to better predict the in vivo response.

So far, only ABX464 (obefazimod) has shown clinical efficacy in the treatment of UC, improving patients at long-term follow-up. The drug was evaluated in phase 2a and 2b studies. ABX464 is a small molecule that indirectly modulates miRNA expression by increasing miR-124 levels in immune cells. Phase 2a studies of this compound in patients with CD have also been initiated [105].

Cell therapy (hucMSC, hAESCs) and modulation of the gut microbiome by miRNA are promising avenues for clinical application. The effect of hucMSC-derived exosomes on the inhibition of the NLRP3 inflammasome appears to be an interesting therapeutic approach that requires further investigation [41]. Therapy of colitis by hAESCs through negative regulation of the TNF/NF-κB pathway may be the basic mechanism of therapy [51].

Regulation of the expression of specific miRNAs may be a new approach not only in the treatment of IBD, but also in diagnostics and monitoring [55], where they can act as prognostic and differentiating biomarkers [56].

## 8. Conclusions

miRNAs play an important role in the pathogenesis and progression of IBD symptoms. Regulating their expression can modulate the inflammatory response and the composition of the gut microbiota, which may help to reduce the severity of the disease. Research is currently underway to determine the actual clinical potential of miRNAs in CD and UC. The work to date is promising, especially for the early diagnosis of IBD, differentiation between CD and UC, monitoring the clinical activity of the disease, and assessing the efficacy of the treatment used in patients.

## Figures and Tables

**Table 1:** Potential actions of miRNA in IBD.

miRNA	Potential Action in IBD	Reference
miR-129-5p	Suppression of the immune response of Th1/Th17 cells;	[37]
	Inhibition of ferroptosis by reducing the expression of ACSL4, COX2, and DMT1; Increasing GPX4 and GSH.	[38]
miR-378a-5p	Epithelial cell regeneration; Wound repair through insulin-like growth factor (IGF) and IGFBP5 signaling; Interaction with two binding sequences within the 3′-UTR of IGFBP5.	[64]
miR-374b-5p	Overexpression of miR-374b-5p increases mRNA and protein levels of IL-17A and IFN-γ; Inhibition of miR-374b-5p significantly decreases mRNA and protein levels of IFN-γ and mRNA expression of IL-17A; Overexpression and inhibition of miR-374b-5p significantly reduce and increase IL-10 mRNA and protein levels, respectively; Mediation of JAK1 and STAT3 pathways.	[36]
miR-106a-5p	Decreasing the luciferase activity of WT-STAT3.	[36]
miR-149	Down-regulation of multiple mRNA gene expression of *IL1A*, *TNF-*α, *CXCl10*, *NOS2* and *CCL2* mediated by NF-κB; promoting AMPK phosphorylation in SW480 cells.	[48]
miR-149-5p	Decreasing expression levels of *NOS2* and *IL1B* in Caco-2 cells; Inhibition of mRNA expression of NOS2, *CXCL17*, *CCL5*, *MMP2* and *MMP12* in SW480 cells.	[48]
miR-143/145	Reducing the expression levels of NOS2 and IL1B.	[64]
miR-369-3p	Reducing BRCC3 protein expression levels; Reducing NLRP3 expression; Reducing ASC adaptor protein; Decreasing caspase-1 activation and release; Decreasing production of the cytokines IL-1β and IL-18.	[45]
let-7b and miR-21	Reduction in *Clostridia Clostridiales Lachnospiraceae Blautia* and *Ruminococcus*; Increasing TNF; Increasing interleukin IL-6 and IL-1β mRNA expression.	[67]
let-7b	Increase in Muc1 expression; Decrease in *Muc5b*.	
miR-21	Increasing levels of the neutrophil marker myeloperoxidase (MPO); Significant increase in paracellular permeability; Increasing expression of *claudin-1*, *-2,* and *-5*; A tendency to reduce *Muc2*.	
miR-23a	Alleviation of colitis; Rapid weight regain after discontinuation of DSS; Decreasing disease activity index; Increasing the number of KI67 + cells per crypt; Increasing levels of regeneration marker genes *Pcna*, *Mcm5*, *Mcm6*, *Ly6a,* and *Lama3* (restoration of epithelial cell polyfunction); Reducing pro-inflammatory cytokines; Chemokines (including TNF-α IFNγ, IL6. IL-1β, and IL12a); Restoring MUC2 levels, increasing ZO-1; Increasing the expression of *Mptx1*, *Occludin,* and *Claudin*-*1*; Inhibition of expression of genes related; Associated with inflammatory damage to intestinal epithelial cells (*Reg3b*, *Reg3g*, *Socs3*, *Cxcl2*, *Hmox1*).	[51]
miR-497	Inhibition of the Wnt/β-catenin pathway in colon tissues and RAW264.7 cells.	[49]
miR149-3p/5p	Decreasing inflammatory cytokines (CRP, IL-6, and TNF-α), serum ESR, serum albumin, and fecal calprotectin.	[63]
miR-192-5p	Increasing autophagy flow in the enterocyte; Strengthening the intestinal barrier.	[60]

miRNA—microRNA; Th1/Th17—T helper 1/T helper 17 cells; ACSL4—Acyl-CoA synthetase long-chain family member 4; COX2—Cyclooxygenase-2; DMT1—Divalent metal transporter 1; GPX4—Glutathione peroxidase 4; GSH—Glutathione; IGF—Insulin-like growth factor; IGFBP5—Insulin-like growth factor-binding protein 5; IL-17A—Interleukin-17A; IFN-γ—Interferon-gamma; JAK1—Janus kinase 1; STAT3—Signal transducer and activator of transcription 3; WT-STAT3—Wild-type Signal transducer and activator of transcription 3; NF-κB—Nuclear factor kappa-light-chain-enhancer of activated B cells; AMPK—AMP-activated protein kinase; NOS2—Nitric oxide synthase 2; Caco-2—Human colon cancer cell line; TNF—Tumor necrosis factor; Muc1—Mucin 1; Muc5b—Mucin 5B; MPO—Myeloperoxidase; IgG—Immunoglobulin G; LPS—Lipopolysaccharide; ZO-1—Zonula occludens-1; *Mptx1*—Metalloproteinase inhibitor 1; *Occludin*—Tight junction protein; *Claudin*-1—Tight junction protein; CRP—C-reactive protein; *Reg3b*—Regenerating islet-derived protein 3 beta; *Reg3g*—Regenerating islet-derived protein 3 gamma; *Socs3*—Suppressor of cytokine signaling 3; *Cxcl2*—C-X-C motif chemokine ligand 2; Hmox1—Heme oxygenase 1; Wnt/β-catenina—Wnt/β-catenin signaling pathway; DSS—Dextran sulfate sodium; PCNA—Proliferating cell nuclear antigen; Mcm5—Minichromosome maintenance protein 5; Mcm6—Minichromosome maintenance protein 6; *Ly6a*—Lymphocyte antigen 6 complex, locus A; *Lama3*—Laminin subunit alpha 3; MUC2—Mucin 2; ZO-1—Zonula occludens-1; Wnt/β-catenina—Wnt/β-catenin signaling pathway.

## Data Availability

Not applicable.
